# Unilateral external fixator and its biomechanical effects in treating different types of femoral fracture: A finite element study with experimental validated model

**DOI:** 10.1016/j.heliyon.2024.e26660

**Published:** 2024-02-17

**Authors:** Aishah Umairah Abd Aziz, Muhammad Imam Ammarullah, Bing Wui Ng, Hong-Seng Gan, Mohammed Rafiq Abdul Kadir, Muhammad Hanif Ramlee

**Affiliations:** a Bone Biomechanics Laboratory (BBL), Department of Biomedical Engineering and Health Sciences, Faculty of Electrical Engineering, Universiti Teknologi Malaysia, Johor Bahru, 81310, Johor, Malaysia; b Bioinspired Devices and Tissue Engineering (BIOINSPIRA) Research Group, Universiti Teknologi Malaysia, Johor Bahru, 81310, Johor, Malaysia; c Department of Mechanics and Aerospace Engineering, College of Engineering, Southern University of Science and Technology, Shenzhen, 518055, Guangdong, China; d Department of Mechanical Engineering, Faculty of Engineering, Universitas Diponegoro, Semarang, 50275, Central Java, Indonesia; e Undip Biomechanics Engineering & Research Centre (UBM-ERC), Universitas Diponegoro, Semarang, 50275, Central Java, Indonesia; f Department of Orthopaedics and Traumatology, Hospital Universiti Kebangsaan Malaysia (HUKM), Cheras, 56000, Federal Territory of Kuala Lumpur, Malaysia; g School of AI and Advanced Computing, XJTLU Entrepreneur College (Taicang), Xi'an Jiaotong-Liverpool University, Suzhou, 215400, Jiangsu, China; h Department of Biomedical Engineering, Faculty of Engineering, Universiti Malaya, Kuala Lumpur, 50603, Federal Territory of Kuala Lumpur, Malaysia

**Keywords:** External fixator, Finite element analysis, Fracture healing, Pin tract infection, Femur

## Abstract

Previous works had successfully demonstrated the clinical effectiveness of unilateral external fixator in treating various types of fracture, ranging from the simple type, such as oblique and transverse fractures, to complex fractures. However, literature that investigated its biomechanical analyses to further justify its efficacy is limited. Therefore, this paper aimed to analyse the stability of unilateral external fixator for treating different types of fracture, including the simple oblique, AO32C3 comminuted, and 20 mm gap transverse fracture. These fractures were reconstructed at the distal diaphysis of the femoral bone and computationally analysed through the finite element method under the stance phase condition. Findings showed a decrease in the fixation stiffness in large gap fracture (645.2 Nmm-1 for oblique and comminuted, while 23.4 Nmm-1 for the gap fracture), which resulted in higher displacement, IFM and stress distribution at the pin bone interface. These unfavourable conditions could consequently increase the risk of delayed union, pin loosening and infection, as well as implant failure. Nevertheless, the stress observed on the fracture surfaces was relatively low and in controlled amount, indicating that bone unity is still allowable in all models. Briefly, the unilateral fixation may provide desirable results in smaller fracture gap, but its usage in larger gap fracture might be alarming. These findings could serve as a guide and insight for surgeons and researchers, especially on the biomechanical stability of fixation in different fracture types and how will it affect bone unity.

## 1 Introduction

External fixation is a well-known surgical treatment used in clinical practice to stabilise bone [1] and soft tissues at a distance from the operative or injury focus [2]. It provides unobstructed access to relevant skeletal and soft tissue structures for their initial assessment and secondary interventions [3]. Its key point includes the ability to stabilise a fracture without the need for open reduction or invasive surgery at the fracture site, which are usually unwanted trauma scenarios. The external fixation is also generally reserved for an open fracture, especially in polytraumatised patients [4]. However, its application has been commonly associated with complications including the malunion [5] and pin tract infection [6]. Concerning femoral fractures, the incidence is reported to range between 10 and 21 per 100,000 per year with the likelyhood in men between the ages of 15–35, while women starting at age 60 [7]. Retrospective study by Alexander et al. stated that the most common fracture seen was mid shaft femoral fracture [8]. Despite their relatively complex appearance, and high pin tract infection risk with up to 90% recorded cases [9], the use of external fixator had shown great success in treating different type of femoral fractures especially.

Clinical finding from Lovisetti et al. [10] also demonstrated a 85%–91% rate of complete union without additional surgery, on top of satisfying functional scores in a practice of peri and intra articular distal femoral fractures treated with circular frame. Ilizarov or circular external fixation that works on the principle of distraction osteogenesis [11] is commonly used as an effective treatment option for complex femoral non-union condition. However, the movement restriction due to its bulky size and complications that come along with it, make a simpler unilateral type of fixator which offers as an alternative solution, whereby this type can provide sufficient stability during the intervention period [12,13]. A prospective study by Agrawal et al. [14] reported that monorail external fixator was the treatment of choice for complex non-union femoral shaft fracture due to its effectiveness and less complication.

Apart from the complex femoral fracture, the previous study had also reported the successful application of unilateral external fixator in treating simple fracture types, such as the oblique and transverse fractures. Kim et al. [15], for instance, had documented their work in using external fixator to treat transverse and oblique femoral fractures in thirteen children. They reported that union was achieved for both in the flexible intramedullary nailing and external fixation treatment, but more cases of overgrowth were seen in the intramedullary nailing as compared to the external fixator. Besides, Testa et al. [16] also revealed the ability of monoaxial external fixator in treating various types of femoral shaft fracture, including open and close types. The three most frequent fractures reported in the study were the 32A3 (transverse) with frequency of 21 cases, followed by 32A2 (oblique) and 32C3 (comminuted) and both with frequency of 13 cases. Briefly, external fixation is a versatile solution for treating various types of fracture, ranging from simple to complex non-union fractures, as well as open and close type fractures. It is also based on the surgeon's personal preference and experience in choosing the type of fixator to be used in the treatment [[17], [18], [19]].

These above-mentioned cases were clinically reported, and the underlying principle and theory is still unclear. The use of external fixator in treating different fracture types need to be justified and supported through well-founded analyses. For that matter, the application of finite element method (FEM) has greatly served its purpose, that is to solve problems of engineering [20], biomedicine, and mathematical physic [21] with complicated geometries [22], conditions [23], loadings [24], and material properties [25], whereby analytical solutions cannot be obtained. The FEM helps in modelling and analysing the external fixator in various circumstances. Sternick et al. [26] evaluated the relation between number of pins in each clamp and rigidity of the external fixation through FEM. Leetha et al. [27] had also implemented FEM to investigate the effects of pin insertion onto the interfragmentary movement. Despite these successful computational analyses, the studies were only investigating into the performance of unilateral fixator on a transverse fracture. As far as the authors were aware, no study was made to analyse the use of external fixator on different fractures, neither the complex AO32C3 type, nor the simpler oblique fracture.

Therefore, this study analyses the stability and effectiveness of external fixator for treating different types of fracture through computational analysis, which is the FEM. It includes three different types of fractures: 1) simple oblique fracture, 2) AO32C3 type comminuted fracture, and 3) 20 mm gap transverse fracture. These fractures represent different level of comminution ranging from low to high comminution. The 20 mm gap fracture was also created to reproduce the fracture site as seen in prior literature [26,28,29]. All fractures were modelled at the diaphysis of a femoral bone and stabilised by an external fixator. Since clinical evidence to prove its success in treating different types of femoral fracture is numerous, unilateral fixator was chosen as a constant, for the type of fixator used in this study. Overall displacement, interfragmentary movement, and stress distribution were observed and evaluated concerning the bone healing and pin tract infection risk. At the end of the study, surgeons and future researchers could briefly look at if they were to apply the fixator on any of the fractures discussed. Also, in the future more work could be explored to improve the external fixation system.

## 2 Materials and methods

In this study, FEM was used for the computational analyses, whereby the problems were remodelled into three-dimensional (3D) shape, resembling closely to the actual situation and analysed accordingly [30]. Von Misses stress, overall displacement, and interfragmentary movement were observed and compared to evaluate the fixation stability as well as its effectiveness in supporting bone healing and minimising infection risk.

### 2.1 Finite element modelling

**Fig. 1:**
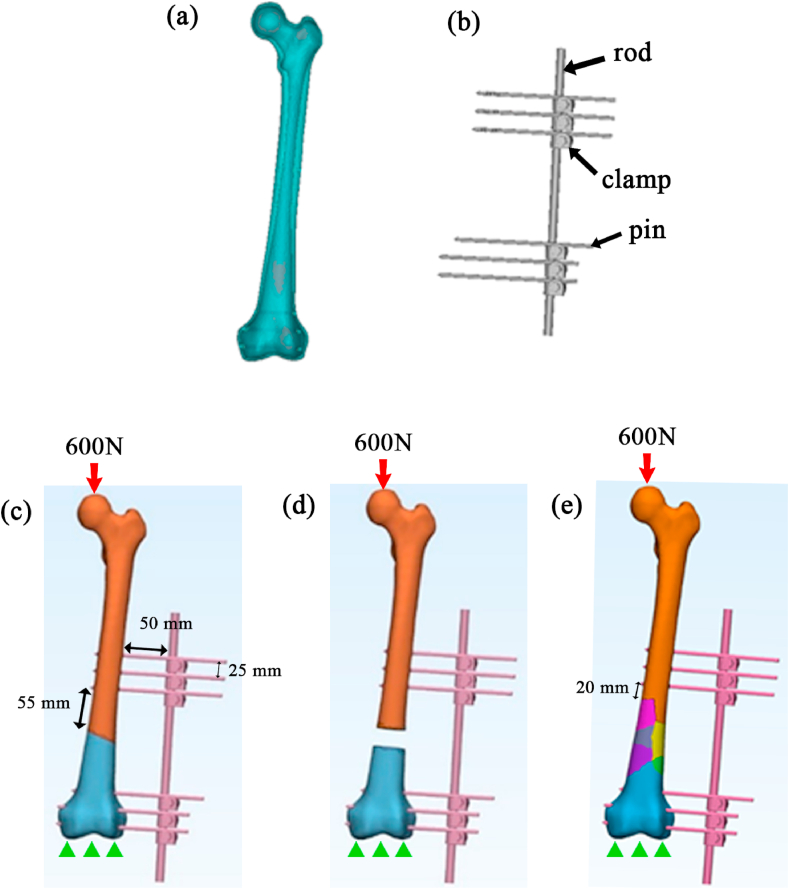
3D model of a (a) healthy bone, (b) Unilateral external fixator, and fitted bone-fixator construct for (c) oblique fracture, (d) 20 mm gap transverse fracture, and (e) complex AO32C3 comminuted fracture, along with the boundary conditions applied.

Computed tomography (CT) datasets with an ethical approval of a healthy 27 years old man with 169 cm in height and 75 kg in weight from Hospital Tunku Ampuan Afzan, Kuantan, Pahang, Malaysia, were used to remodel the femoral bone. The usage of the CT datasets in this study was approved by the medical ethical committees from Hospital Tengku Ampuan Afzan, Kuantan, Pahang, Malaysia as shown in appendix A (no: versi2.0tarikh15Feb2008), where identification of patient has been made anonymous. The image of left femur was segmented and reconstructed into a 3D model by using the Mimics software (Materialise Technologies, Leuven, Belgium) [31]. Two bone layers were considered and differentiated based on Hounsfield Unit (HU). It was 750 HU to 3071 HU for the cortical layer and 200 HU to 750 HU for the cancellous [32]. The healthy bone model (Fig. 1 (a)) was then cut according to the desired fracture type, which were the oblique (Fig. 1 (c)), transverse with 20 mm gap (Fig. 1 (d)), and complex A032C3 comminuted fracture (Fig. 1 (e)) in 3-Matic software (Materialise Technologies, Leuven, Belgium). All fractures were made at the distal diaphysis of bone. As for the unilateral external fixator, it was remodelled into a 3D shape by using reverse engineering method in SolidWorks CAD software (Dassault System SolidWorks Corp., Waltham, USA) [33]. It consisted of a 11 mm diameter rod, six screws with diameter of 5 mm each and six clamps to fix the screws and rod [33]. The configuration of fixator is shown in Fig. 1(b). Then, the external fixator and fractured bone models were fitted together in the 3-Matic models (Fig. 1(c)–. (d), and Fig. 1 (e)). For every model, the bone to rod distance was set at 50 mm and 25 mm for the pin to pin distance. Meanwhile, distance between inner most pin and the nearest fracture line for oblique and gap fracture was set to 55 mm and 20 mm for the comminuted fracture.

In the same software, all models were meshed into the first order tetrahedral elements [34]. To ensure reliability and consistency of results, mesh convergence analyses [35] were done individually for femur and fixator. Based on results, element size for the bone was set at 4.5 mm and 1.0 mm for fixator. Table 1 details the number of nodes and elements for each model.

**Table 1:** Convergence study details of the femur model.

Component	Total number of nodes	Total number of elements
External fixator	484,965	128,562
Femur (oblique)	29,025	160,794
Femur (20 mm gap transverse)	45,551	208,346
Femur (complex AO32C3)	33,138	216,975

### 2.2 Validation of FE model

**Fig. 2:**
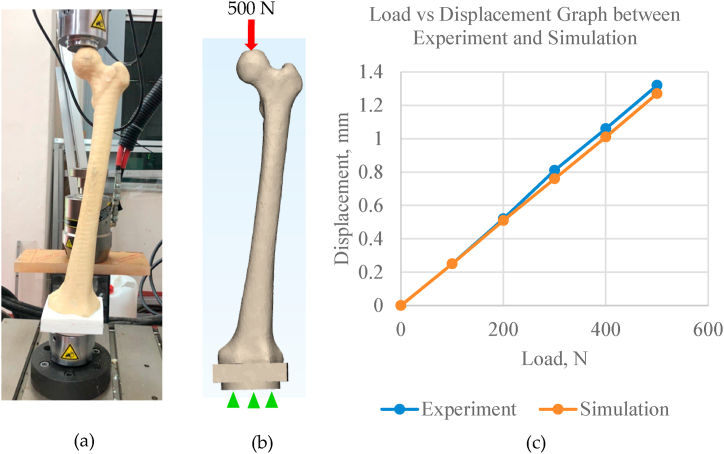
Validation test where the (a) femoral bone was compressed under axial compression, mimicking the (b) 3D FE femoral bone model, and the (c) graphical presentation of the results describing the correlation of the two tests.

On top of that, the FE model of femur was also validated experimentally to increase reliability of the analyses [36]. To ensure it is a one-to-one validation, the 3D femoral model was printed out by using a 3D printer (Zortrax, Poland). Polylactic acid (PLA) filament with 1.75 mm diameter was used and supplied by the Shenzhen Esun Industrial Co., Ltd. Following that, synthetic bone from polyurethane (PU) was fabricated based on printed bone sample [30]. The printed bone model was used as a template for the moulding process where the bone mould was made from silicone rubber (Moldmaker RTV Silicone Rubber). Then, synthetic bones made from PU were casted from the mould, having the same shape and morphology with the model used in the simulation [[37], [38], [39]]. As shown in Fig. 2 (a), the synthetic bone was then tested under 500 N axial compression by using Instron 8874 universal testing machine (UTM) (Instron Ltd, High Wycombe, UK). At the same time, the FE analysis was also conducted on the FE femoral model with all properties and boundary conditions being set to represent the compression test (Fig. 2(b)). Displacement was observed for both tests and the accuracy of prediction was expressed by the coefficient of linear regression (r2). The average percentage difference calculated was only 2.83%, with r2 = 0.999, as shown in the graph (Fig. 2(c)).

### 2.3 Finite element analyses

All models were assumed as isotropic, homogenous, and linearly elastic materials [40] with material properties set as in Table 2. All external fixator components were generalized into one type of material which is the stainless steel with material properties stated in the table. For the contact modelling, fully bonded contact was set between cortical and cancellous layer, while partially bonded for the contact between femoral bone and external fixator [41]. The friction coefficient was set to 0.3 [42]. This study was based on the single-legged stance phase of a human being [43]. Therefore, to represent the situation, a 600 N axial load was applied onto the most proximal point of the bone [44], whereas at the distal end of femur was fixed in all directions [45]. The point load represented a hip joint force, whereby it simplified the hip muscle reaction, as widely used in prior studies [46]. Fig. 1 illustrates the boundary conditions for all models.

**Table 2:** Convergence study details of the femur model.

Component	Young's modulus (MPa)	Poisson ratio (−)
Cortical layer	16,500 [47]	0.3 [48]
Cancellous layer	150 [49]	0.3 [50]
External fixator	190,000 [51]	0.3 [51]

## 3 Results

### 3.1 Displacement and IFM

**Fig. 3:**
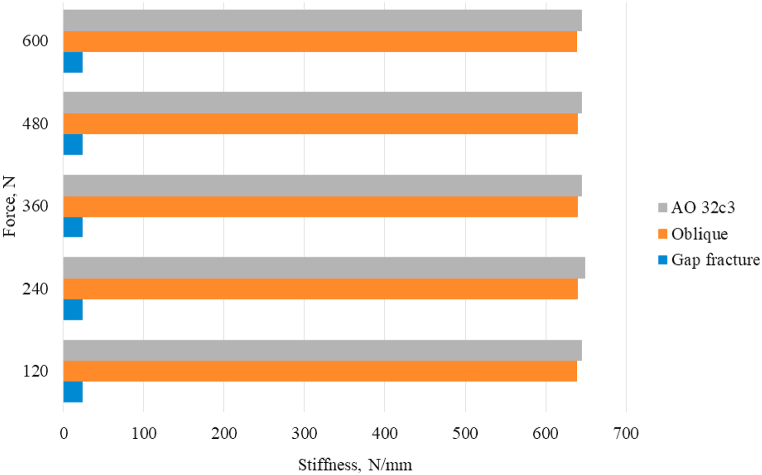
Stiffness calculated for the three different fracture type.

Displacement of the fixator as a whole was smaller in oblique and comminuted fractures (0.25 and 0.26 mm, respectively), but increased greatly in the 20 mm gap fracture, with magnitude of 11.40 mm. Similarly for the displacement on the bone, whereby only 0.93 mm displacement was observed on the oblique and comminuted fracture models, while the gap fracture showed a 25.60 mm displacement. There was up to 186 % difference calculated between the two reported values. The stiffness value calculated were 645.2 Nmm-1 for oblique and comminuted fractures and 23.4 Nmm-1 for gap fracture (Fig. 3).

**Fig. 4:**
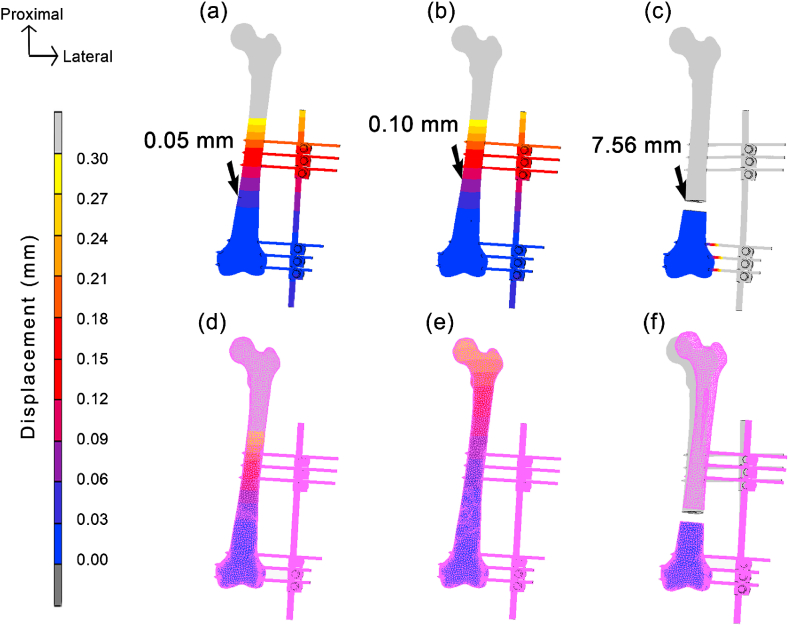
Displacement and IFM for (a) oblique, (b) comminuted, and (c) gap fracture, as well as the deformation for (d) oblique, (e) comminuted, and (f) gap fracture.

Fig. 4 (a – f) illustrates the displacement between three types of fracture, as well as the deformation, whereby the pink sketch indicated the original shape of model. It can be clearly observed that the 20 mm gap fracture deformed the most, which corresponded to its displacement value, and recorded the highest in both fixator and bone. Besides, IFM was also observed at the fracture surface of proximal bone fragment. The oblique fracture showed 0.05 mm movement, followed by comminuted fracture with a peak value of 0.10 mm, and finally the highest movement in gap fracture, which was 7.56 mm.

### 3.2 Stress distribution

**Fig. 5:**
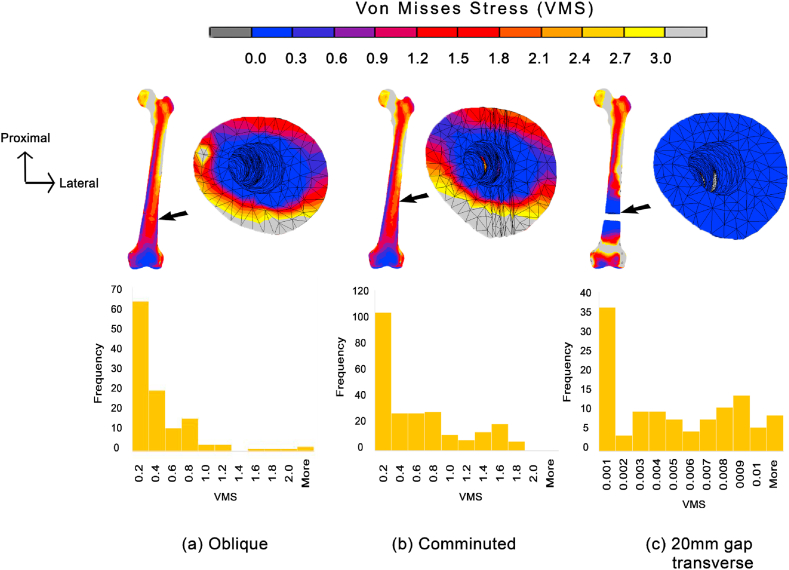
Stress contour and histogram of VMS distribution specifically on the fracture gap between the three types of fracture for (a) Oblique, (b) Comminuted, and (c) 20 mm gap transverse.

Apart from that, Von Misses stress (VMS) was also observed. The VMS was first observed on the fracture surface of proximal bone fragment. It was found that the gap fracture produced the least stress value with only 0.012 MPa at maximum. The histogram of the stress distribution on Fig. 5(a–c) exhibited the highest frequency of elements are of stress lower than 0.001 MPa. On the contrary, both oblique and comminuted fractures showed almost similar peak values, which were 2.091 MPa and 1.759 MPa, respectively. The histogram also showed that the highest frequency of stress was found in elements with less than 0.2 MPa for the two types of fracture. Higher frequency of elements with more than 0.2 MPa was also found in the comminuted fracture as compared to the oblique.

**Fig. 6:**
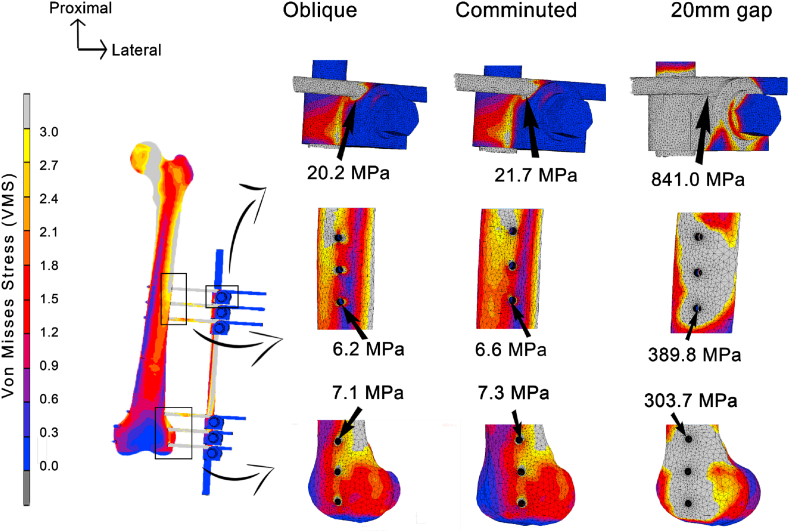
Stress contour between the three types of fracture.

As for the full models, the stress contour plot is shown in Fig. 6. For the fixator frame, peak stresses were found at the first pin clamp interface for all models. The value showed a big difference in the 20 mm gap fracture as compared to the other two models. It was 841.0 MPa in the 20 mm gap fracture, followed by 21.7 MPa in the comminuted fracture, and 20.2 MPa in the oblique type. The same trend was also seen on bone models, specifically at the nearest pin-bone interface to the fracture line. It was the highest in the gap fracture (389.8 MPa in proximal and 303.7 MPa in distal bone), succeeding by the comminuted fracture (6.6 MPa for proximal and 7.3 MPa for distal fragment), and oblique (6.2 MPa in proximal and 7.1 MPa in distal bone). Although there was great difference in the gap fracture, the oblique and comminuted fractures produced almost similar peak stress magnitudes with only a maximum of 7.2% difference. These high VMS values were also seen to concentrate at the lateral side of bone, which was the entry hole of pin.

## 4 Discussion

The AO/OTA classification has categorised various types of femoral fracture, including the simple oblique, fragmented wedges, and complex irregular fracture [52]. Commonly, internal fixation is used in treating these fractures, but external fixations will be applied if the situation is impossible or in-advisable, such as in open fracture cases [53]. For example, Lawal et al. [54] reported their successful management of open diaphyseal fractures by using the monolateral external fixators for long bones, including the femur. Agrawal et al. [14] also claimed that for treating complex non-union femoral shaft fracture, monorail external fixator was an effective treatment option due to its comparable functional outcome as in any other treatment options. Nonetheless, there were debates on the use of external fixator for treating various types of femoral fracture, whether it was able to provide sufficient stiffness in supporting the bone unity. There was lack of justifications on its mechanical stability, and how it affected the bone healing process and complication risk. As far as the author is aware, there are limited studies which reported on the effect of external fixator in different fracture types, especially on femoral bone. Therefore, as a pilot study, these computational analyses were conducted to compare the stability of unilateral external fixation in different types of fracture, including simple oblique fracture, AO32C3 type comminuted fracture, and transverse gap fracture.

In FEM implementation, it is always important to use validated models [55]. Therefore, through the reverse engineering method, the bone model was reconstructed in detail based on a correct anatomical structure, ensuring its accuracy to give reliable results [56]. Validation steps were taken to further increase confidence, where it can be defined as a process, whereby the 3D FE model predictions are compared to the experimental data in order to determine the modelling error [57]. It informs the investigator if a given FE model is the right model and an accurate representation of the ‘real world’. For instance, a study by Macleod et al. [58] validated the open-source model of the femoral bone for use in three commonly used FE packages: Abaqus, Ansys, and Febio. They predicted the strain measurement on the fourth-generation Sawbones composite femurs and found that all solvers produced excellent agreement. Ebrahimi et al. [59] also validated their femoral bone model in their study, which was based on the composite femur (Sawbones). They tested the bone experimentally and computationally, and eventually compared the results, which also showed an excellent agreement. Therefore, following the same concept, the 3D bone model used in this study was compared to the experimental test on the synthetic bone, as described earlier. Displacement was observed and compared between the two tests, and it showed only a small percentage error. This conformed the validity of femoral bone model, and thus the following analyses were proceeded.

Generally, the oblique and comminuted fractures had shown significant distinct results as compared to the 20 mm gap fracture. Aside from the 20 mm gap fracture which portrayed a high displacement, both oblique and comminuted fractures had shown nearly equivalent and low displacement for bone and fixator. It illustrated that the fixation system was less stiff in the large gap fracture, in contrast to the small gaps. Comparable results were found in a study by Kouassi et al. [60] whereby the fixation stiffness was greater in oblique as compared to the gap fracture with axial stiffness values of 119.7 Nmm-1, and 71.8 Nmm-1, respectively. Low stiffness depicted low fixation stability, which in turn, may disrupt the healing process of the bone as it needs a stabilised and controlled mechanical environment for the formation of callus in bridging the bone fragments [61]. Accordingly, the results showed a similar shift on the IFM, whereby the value was much smaller in the oblique and comminuted fractures than in the gap fracture. As in the experimental study by Claes et al. [62], the small gaps fracture produced higher stiffness with lower interfragmentary movement as compared to the large gap fracture. A less than 1 mm movement was needed for the bone healing to take place [26]. The observed values were much smaller and within the allowable range for both oblique and comminuted fractures, but greatly increased and over the safe limit for the gap fracture. This excessive movement was not preferable as it may inhibit the callus formation, causing a delayed fracture healing [63].

Similar trend was also seen on the stress distribution, whereby the peak value was extremely high in the gap fracture, in fact the peak stress found on the fixator frame was beyond the yield strength of stainless steel (515 MPa [64]). As it had surpassed the maximum allowable stress, the material will start to deform plastically causing implant failure. The peak stress found on the bone of gap fracture, specifically at the pin-bone interface, was also beyond the yield strength (200 MPa for bone [65]), and thus increasing the risk of refracture. On the other hand, the oblique and comminuted fractures demonstrated an inverse situation, whereby the peak stresses were below the limits and nearly had the same value in both bone and fixator. It indicated that both fixations were in safe mode, and fixation failure was avoidable. As increasing stress causes an increasing risk of pin loosening and infection [66], the 20 mm gap fracture was concluded to be most likely to experience those complications as compared to the other two fracture types. Besides, stress distribution was also observed on the fractured surface of proximal fragment. Despite the low stress shown by oblique and comminuted fractures (2.091 MPa and 1.759 MPa, respectively), the gap fracture produced a significant smaller value with a maximum value of only 0.012 MPa. Low stress at the fracture site along with good blood supply is actually needed as it could encourage bone formation [67], while high stress could interfere with callus formation [68]. The low stress value shown by all fracture types suggested that the healing process is allowable and fracture union can be achieved.

Generally, it was interesting to note that both oblique and comminuted fractures were made in perfect fit, approximately 0.1 mm gap with partially bonded contact modelling, while the 20 mm gap fracture was made as ‘no contact’ model. The large gap between the bone fragments indeed played a significant role. Mechanically, the degree of IFM depends on the type and stiffness of fracture fixation [63]. Larger gap fracture decreases the bending stiffness of fixation, resulting in higher IFM. This was in agreement to prior study by Claes et al. [62] who found lower movement in smaller fracture gap, and thus producing a higher bending stiffness. Preclinical investigation by Meeson et al. [69] also showed a similar trend, whereby predominant delayed union was demonstrated in 1.5 mm gap as compared to the previously published studies that used the same fixator for treating a 0.5 mm fracture gap. Biologically, these delayed fracture healing could be due to limited ability of vascularisation that was needed for callus formation, whereby without it, only fibrocartilaginous tissue can develop, leading to a pseudarthrosis [70].

Based on these biomechanical evaluations, it can be concluded that the use of unilateral external fixator could be fatal in large gap fracture, yet encouraging and favourable in smaller gaps. It is not advisable to choose the unilateral external fixator for relatively large fracture gaps during the intervention period. Other structure of external fixator, such as multiplanar configurations, could be the option for treating gap fracture as claimed by Kouassi et al. [60] in their study. The use of external fixator alone as the primary treatment shall be revised and more precautions should be taken into account. In this study, there were a few assumptions and simplifications made due to the inevitable limitations. As such, an absolute decision was made on the bone materials to improve the accuracy within a limited range of sources. The assumptions and simplifications were not only made in this particular study, but also it should be noticed that those actions were also considered by many other scholars in simulating bone and implant structures with an acceptable result. First, the bone properties were assumed as homogenous, isotropic, and linearly elastic, despite the realistically, non-homogenous and orthotropic properties [71]. Besides, the modulus and strength set for the bone was also generalized into one value, whereby it might be over-estimated, especially for the cancellous bone [72]. Nevertheless, these assumptions were acceptable as many previous studies had successfully applied them due to the complexity of model and limited computer resources [73], besides the insignificant difference in stress/strain predictions between the use of orthotropic versus isotropic bone material properties [74].

As there were various loading conditions used in previous literature in analysing femoral bone, assumption was also made on what load to be applied. Regardless, it should be noted that an appropriate recommendation regarding weight bearing is an important clinical issue [75]. Therefore, in this study, the load applied was limited to the stance phase of a gait cycle, mimicking the early weight bearing of a patient upon the fixation application. Some studies applied half body weight load on the femoral head [76] while some others used every muscle and ligament effects on the femur [77]. Since it was realistic and resource-wise, the 600 N load on femoral head was chosen as it sum up the hip muscle reaction as demonstrated by, besides being widely used in previous studies with reasonable and acceptable results.

## 5 Conclusions

Unilateral external fixation may provide desirable results in smaller fracture gaps, but its usage in larger gap fracture might be alarming. As such, for the gap fracture, eventhough it produced low stress at the fracture surface that may indicate that bone unity is allowable, the fixation stiffness was decreased, resulting in higher IFM and stress distribution at the pin-bone interface. These consequences are unfavourable as they increase the risk of delayed union, pin loosening and infection, as well as implant failure. The results from this study could be a guide and insight on the biomechanical stability of the fixation in different types of fracture and how it affect bone unity. Although it was clinically accepted that external fixator could provide positive outcomes for treating various types of femoral fracture, it is important to always take extra precautions during decision-making.

## Additional information

No additional information is available for this paper.

## Funding statement

This work was supported by the 10.13039/501100005417Universiti Teknologi Malaysia (UTM) (grant number 04M13, 4J583 and 04M22).

## Institutional review board statement

Not applicable.

## Informed consent statement

Not applicable.

## Data availability statement

The data presented in this study are available on request from the corresponding author.

## CRediT authorship contribution statement

**Aishah Umairah Abd Aziz:** Conceptualization, Data curation, Formal analysis, Funding acquisition, Investigation, Methodology, Project administration, Resources, Software, Supervision, Validation, Visualization, Writing – original draft, Writing – review & editing. **Muhammad Imam Ammarullah:** Conceptualization, Data curation, Formal analysis, Funding acquisition, Investigation, Methodology, Project administration, Resources, Software, Supervision, Validation, Visualization, Writing – original draft, Writing – review & editing. **Ng Bing Wui:** Conceptualization, Data curation, Formal analysis, Funding acquisition, Investigation, Methodology, Project administration, Resources, Software, Supervision, Validation, Visualization, Writing – original draft, Writing – review & editing. **Hong-Seng Gan:** Conceptualization, Data curation, Formal analysis, Funding acquisition, Investigation, Methodology, Project administration, Resources, Software, Supervision, Validation, Visualization, Writing – original draft, Writing – review & editing. **Mohammed Rafiq Abdul Kadir:** Conceptualization, Data curation, Formal analysis, Funding acquisition, Investigation, Methodology, Project administration, Resources, Software, Supervision, Validation, Visualization, Writing – original draft, Writing – review & editing. **Muhammad Hanif Ramlee:** Writing – review & editing, Project administration, Conceptualization, Data curation, Formal analysis, Funding acquisition, Investigation, Methodology, Resources, Software, Supervision, Validation, Visualization, Writing – original draft.

## Declaration of competing interest

The authors declare that they have no known competing financial interests or personal relationships that could have appeared to influence the work reported in this paper.
